# Ipecac Treatment of Dysentery

**Published:** 1915-02

**Authors:** 


**Ipecac Treatment of Dysentery** used by Helvetius as early as 1686 when he advertised it by bill boards in Paris. ("Ec. Med. Jour.," Jan. 1915.) Now explained that emetine is a specific amoebacide, not lethal to other micro-organisms.

